# Fighting Enteroviral Infections to Prevent Type 1 Diabetes

**DOI:** 10.3390/microorganisms10040768

**Published:** 2022-04-01

**Authors:** Magloire Pandoua Nekoua, Ambroise Mercier, Abdulaziz Alhazmi, Famara Sane, Enagnon Kazali Alidjinou, Didier Hober

**Affiliations:** 1 Laboratoire de Virologie ULR3610, Université de Lille, CHU Lille, 59000 Lille, France; magloire-pandoua.nekoua@univ-lille.fr (M.P.N.); ambroise.mercier.etu@univ-lille.fr (A.M.); abalhazmi@jazanu.edu.sa (A.A.); famara.sane@chru-lille.fr (F.S.); enagnonkazali.alidjinou@chru-lille.fr (E.K.A.); 2 Microbiology and Parasitology Department, College of Medicine, Jazan University, Jazan 82911, Saudi Arabia

**Keywords:** enteroviruses, coxsackievirus, antiviral, vaccine, type 1 diabetes

## Abstract

Enteroviruses (EVs), especially coxsackieviruses B (CVB), are believed to trigger or accelerate islet autoimmunity in genetically susceptible individuals that results in type 1 diabetes (T1D). Therefore, strategies are needed to fight against EV infections. There are no approved antiviral drugs currently available, but various antiviral drugs targeting viral or host cell proteins and vaccines have recently shown potential to combat CVB infections and may be used as new therapeutic strategies to prevent or reduce the risk of T1D and/or preserve β-cell function among patients with islet autoantibodies or T1D.

## 1. Introduction

Enteroviruses (EVs) are small (25 to 30 nm diameter) non-enveloped, positive-sense single-stranded RNA genome viruses which belong to the *Picornaviridae* family [1]. The genus Enterovirus comprises 15 species, seven of which infect humans (*Enterovirus A–D* and *Rhinovirus A–C*) and over 250 serologically distinct viruses. *Enterovirus A–D* include more than 110 serotypes, the best known of which are the polioviruses, enterovirus A71, echoviruses, coxsackieviruses A and coxsackieviruses B [2]. These viruses are ubiquitous in the world and are transmitted mainly by the fecal-oral or respiratory routes following a seasonal pattern. EVs infections occur often during summer and early fall in temperate zones while they are constant throughout the year in tropical zones [3].

They replicate primarily in the gastrointestinal tract or in the upper respiratory tract and can spread to various target organs through the lymphatic system and the bloodstream [3]. They are cytolytic viruses, but they can establish persistent infections in vitro as well as in vivo [4]. Most enteroviral infections remain asymptomatic but they are responsible for numerous clinical signs of varying severity depending on infection site or virus serotype. Enterovirus infections can cause relatively mild symptoms such as common cold, fever, and skin lesions (not requiring hospitalization) or severe acute conditions such as meningitis, hepatitis, encephalitis, myocarditis, pancreatitis, hand, foot, and mouth disease, pericarditis, neonatal sepsis, and acute flaccid paralysis [5,6]. In addition to acute diseases, EVs have also been associated with chronic diseases such as type 1 diabetes (T1D).

Several epidemiological, clinical, and experimental data support the hypothesis that CVB are the most incriminated environmental factors linked to the development of islet autoimmunity or onset and progression of T1D [7,8]. Despite the high prevalence of non-polio EVs in the world [9], there are no approved antiviral drugs available for clinical use to treat EV infections. However, antiviral drugs and vaccines have recently shown potential to combat EV serotypes and may be used as new strategies to fight EV infections to prevent or reduce the risk of T1D and/or preserve β cells function in susceptible individuals. In this article, we discuss ongoing scientific research projects evaluating antiviral strategies against CVB and provide an overview of relevant new avenues to combat CVB infection for preventing T1D.

## 2. Strategies to Fight against Coxsackieviruses B

The EVs genome is approximately 7400 bases and contains a single open reading frame flanked by an untranslated region at 5′ and 3′ ends [7]. CVB replication cycle consists of different steps. Briefly, the viral particle binds to a specific cell surface receptor, mainly the coxsackie and adenovirus receptor (CAR) and/or to a co-receptor such as decay accelerating factor (DAF or CD55). The virus-cellular receptor interaction leads to virus internalization into cell through different endocytosis pathway depending on the serotype and cell type [10]. After virus internalization, receptor binding and/or low endosomal pH induce conformational changes in the viral capsid that result in the release of viral genome into the cytoplasm through the endosome membrane (virus uncoating) [11]. A translocation of the viral RNA directly into the cytoplasm through a membrane channel described for polioviruses cannot be excluded for CVB [12]. Then, viral RNA is translated into a viral polyprotein which is proteolytically processed in structural proteins (VP1-4) and seven non-structural proteins (2A^pro^, 2B, 2C, 3A, 3B, 3C^pro^, and 3D^pol^) [7]. EV infection induces replication vesicles on which positively and negatively stranded viral RNAs are produced with the help of nonstructural proteins (especially 3D, but also 2B, 2C, and 3AB). Positive viral RNA encapsidates with structural proteins then new viral particles are released by the lysis of infected cell or by non-lytic mechanisms [13]. Viral proteins or host factors involved in virus replication can potentially be the target of antiviral drugs.

### 2.1. Antiviral Agents Targeting Viral Proteins

Pleconaril is one of the most studied antiviral drugs that was initially developed for the treatment of common cold or prevention of asthma exacerbation due to picornavirus infections [14]. Pleconaril binds the virus capsid and inhibits the attachment to the cellular receptors and the uncoating and consequently prevent the release of the viral RNA. It was rejected by the U.S. Food and Drug Administration (FAD) due to the emergence of resistant virus [15]. Nevertheless, in vitro and in vivo antiviral activities of pleconaril have been reported against some EVs such as CVB3 or CVB4 [16,17,18]. However, some authors also reported recently complete inefficacy of this molecule against CVB3 in vitro [19]. Ribavirin is a nucleoside analog approved by FDA as an antiviral drug to treat chronic hepatitis C virus infections in combination with peginterferon alfa-2a [20] or to treat severe respiratory syncytial virus infections [21] and its antiviral effects against EVs have also been reported [22]. Indeed, ribavirin can impair virus replication through induction of lethal mutagenesis by interacting with the viral protein 3D^pol^ (a RNA-dependent RNA polymerase) [22]. Antiviral effect of ribavirin against CVB3 replication was shown to be enhanced by human IFN-α [23]. Remdesivir is a mono-phosphoramidate adenosine analog prodrug which can inhibit CVB3 RNA synthesis [24]. Umifenovir (trade name Arbidol), an antiviral chemical molecule known to inhibit various human respiratory RNA viruses by abrogating virus-endosome fusion [25] has shown antiviral activity against CVB5 in vitro and in vivo as well [26].

Some repurposed drugs such as fluoxetine (a selective serotonin reuptake inhibitor used as antidepressant drug) targeting non-structural viral 2C protein [27,28,29], itraconazole (an antifungal drug) targeting the non-structural viral protein 3A [30] or emetine (antiprotozoal drug) with inhibitory effect on IRES activity [31], were found to have anti-CVB activity. There is growing interest in studying antiviral activity of repurposed drugs as they may quickly enter into clinical trials. Fluoxetine can inhibit CVB4 replication in vitro in models of acute and persistent infection of pancreatic cells (human and murine) [32,33] and in vivo in organs of infected CD-1 mice [33]. Pleconaril and hizentra (a human immunoglobulin concentrate containing IgG neutralizing antibodies against EVs) may also inhibit persistent infection of pancreatic cells with CVB1 [34]. However, the emergence of fluoxetine-resistant variants has been observed during treatment of pancreatic cell cultures persistently infected with CVB4 [35]. Compound 17 is a benzene sulfonamide derivative that can inhibit the replication of CVB1, CVB3 and CVB6 by targeting a pocket, formed by viral proteins VP1 and VP3, which is a key region for conformational changes required for RNA release [36]. Favipiravir is a viral polymerase inhibitor that is effective against all CVB serotypes in vitro except for CVB2 [19].

### 2.2. Targeting Host Proteins to Fight against Coxsackieviruses B

Enviroxime, PIK93, and GW5074 are kinase inhibitors that can inhibit CVB replication by targeting the host factor, phosphatidylinositol 4-kinase type IIIβ, involved in the positive-strand RNA synthesis [37,38]. Enviroxime is also able to inhibit the persistent infection of pancreatic cells by CVB1 [34]. However, CVB3 may acquire resistance against these antiviral compounds through mutation in 3A protein [38]. Itraconazole may also impede CVB3 RNA replication by targeting the cellular oxysterol-binding protein (OSBP) and OSBP-related protein 4 (ORP4), involved in transport of cholesterol and phosphatidylinositol-4 phosphate (PI4P) and in cell signaling, which disturb the formation of virus replication vesicles [39,40]. Amiloride and 5-(N-ethyl-N-isopropyl)amiloride (EIPA) (blockers of the epithelial Na^+^ channel and the cellular Na^+^/H^+^ exchanger) strongly inhibit CVB3 RNA replication but emergence of amiloride-resistant mutants with amino acid substitutions within the 3D^pol^ was reported [41]. Golgicide A can drastically inhibit CVB3 RNA replication by targeting Golgi-specific Brefeldin A-resistance factor 1 (GBF1), a cellular regulator involved in the transport between the endoplasmic reticulum and Golgi vesicles [42]. Hsp90 (ATP-dependent molecular chaperone) inhibitors such as geldanamycin have been reported to impair the replication of CVB3 [43].

### 2.3. Coxsackieviruses B Vaccines

Effective vaccines have been developed against EVs such as polioviruses and EV-A71 [44,45] but vaccines against CVB are still in the experimental phase or in clinical trials. Recently, a highly immunogenic formalin-inactivated CVB1 vaccine has been able to provide protection against acute CVB1 infection in NOD mice and against CVB1-induced diabetes in transgenic SOCS1-tg mice [46]. A multivalent inactivated vaccine comprising all CVB serotypes (CVB1-6) has been shown to have a good safety profile and to induce high production of neutralizing antibodies in mouse models as well as in nonhuman primates (rhesus macaques). It can also protect Balb/c mice from acute CVB3 infections of the heart and prevent the development of CVB-induced diabetes in SOCS-1-tg mice [47]. This vaccine named PRV-101 vaccine is currently used in a randomized clinical trial (NCT04690426) [48]. An alternative vaccine strategy based on the use of virus-like particles has been developed for CVB1, 3, and 4 and it induces a strong immune response in mice [49,50,51,52].

## 3. Fighting Enteroviruses Opens Perspectives for the Prevention of Type 1 Diabetes

Type 1 diabetes (T1D) is an autoimmune endocrinological disorder characterized by chronic hyperglycemia caused by selective destruction or dysfunction of β cells in the pancreas leading to severe endogenous insulin deficiency. According to a recent meta-analysis the incidence of T1D is currently estimated to be 15 per 100,000 people per year and the prevalence was 9.5% worldwide [53] with an annual incidence rate rapidly increasing by 3–4% over the last 25 years in many countries especially in children and adolescents [54]. Patients with T1D require life-long treatment with daily injections of exogenous insulin and have an increased risk of developing vascular and neurological complications resulting in higher medical care costs and reduced well-being and life expectancy of these patients. Despite the advances of insulin therapy, many patients with T1D do not reach the glycemic control necessary to prevent the progression of diabetes complications. Therefore, new treatments and technologies for diabetes care and management such as pancreas, islet and stem cells transplantation, immunotherapy have been developed with the hope of improving β-cell function or delaying the clinical onset of T1D [55]. However, disease prevention strategies remain a major goal in the diabetes research field.

The precise etiology and the mechanisms that trigger autoimmunity against islet antigens are not fully understood but growing evidence support that the disease process is initiated by environmental factors in genetically susceptible individuals [56]. Several epidemiological, clinical and experimental data support the hypothesis that enteroviruses and especially CVB are the most incriminated environmental factors linked to the development of islet autoimmunity or onset and progression of T1D [7,8]. Indeed, markers of enteroviral infection (protein, RNA or antibodies) are more often detected in the saliva, serum, monocytes, gut mucosa and pancreas of patients with T1D [57,58,59,60,61,62,63,64]. The association between the presence of these markers of infection and the appearance of islet autoimmunity or T1D was statistically confirmed by two case–control meta-analyses including 4448 and 5921 participants, respectively [65,66]. Various mechanisms are suggested to trigger CVB-induced autoimmunity against islet antigens including virus-induced inflammation, bystander activation of preexisting autoreactive T cells, disturbance of tolerance, and viral persistence [4,7,8,67].

It has been estimated that 80% of T1D could be prevented or cured by eliminating the effect of diabetogenic viruses [68]. Therefore, strategies are needed to fight against EV infections for preventing or reducing the risk of T1D in susceptible individuals and/or preserve β-cell function among infected and newly diagnosed T1D patients. In this light, a six-month treatment with the combination of pleconaril and ribavirin currently aims to eliminate persistent EV infection in the pancreas of newly diagnosed T1D patients in a randomized clinical trial (NCT04838145) [69]. Molecules such as Hizentra, enviroxime, favipiravir have shown their efficacy against CVBs in vitro and within the limits of their recommended therapeutic serum concentrations [19], which can be an asset for future clinical phases.

Research into alternative treatments based on natural products from bacteria and/or their metabolites and plant extracts may provide an alternative to synthetic molecules. For example, it has been reported that lipoproteins from bifidobacteria and *Lactobacillus plantarum* can inhibit CVB4 infection in vitro [70,71]. Few studies have evaluated the effects of plant extracts against EVs [72] and this avenue deserves to be explored. It has been shown that aqueous extracts of *Syzygium brazzavillense* can inhibit CVB infection of cells in vitro [73].

The high mutation rates of RNA viruses such as CVB lead to the emergence of resistant variants [74,75] often after treatment with some antiviral agents targeting viral proteins and it is crucial to define new approaches to reduce the effects of these viruses in the pathogenesis of T1D. Indeed, microRNAs (miRNAs), regulators of cellular gene expression, can be deregulated in β cells by CVB infections and may play a role in the development of islet autoimmunity and T1D [76,77,78]. These cellular factors can inhibit or sometimes increase virus replication by targeting the viral genome or the host genes [79]. An antiviral strategy would be to inhibit pathogenic miRNAs as in the case of hepatitis C virus [80,81] or to deliver artificial miRNAs that inhibit viral replication in infected cells [82]. Endogenous human retroviruses envelope protein (HERV-W-Env) is highly expressed in patients with T1D and can be activated in human primary pancreatic ductal cells and macrophages infected with CVB4 [83,84]. The pathogenic effects of these endogenous factors has been suggested to play a role in the pathogenesis of T1D [85] and could be a potential target in disease prevention.

Interferon (IFN) response markers are often identified in islets of T1D patients and are associated with EV persistent infection [86,87,88]. Although type I IFNs, in particular IFN-α, confer to pancreatic islets a decreased permissiveness to CVB infection [89,90], they also have deleterious effects on pancreatic β cells (including endoplasmic reticulum stress, impairment of insulin secretion, and apoptosis) which can play a role in the development of T1D [86,87,91,92]. In a recent study, baricitinib, an oral inhibitor of Janus kinases 1 and 2, was able to significantly reduce in vitro deleterious effect of IFN-α on human β cells and islets [93]. The antagonistic effect of this molecule opens up prospects for prevention of development of T1D in individuals potentially carrying IFN response markers and/or a persistent EV infection.

**Figure 1:**
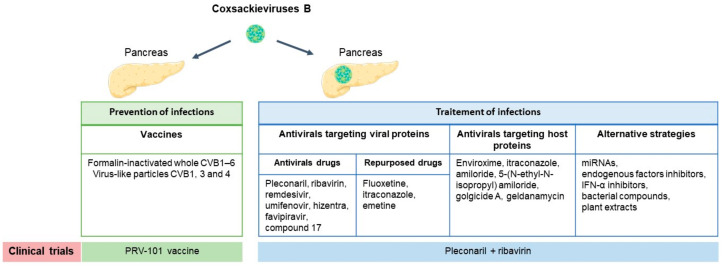
Antiviral strategies for the prevention or treatment of type 1 diabetes (T1D) induced by enteroviruses (EVs) infections, especially coxsackieviruses B (CVB). Vaccines against CVB administered to newborns or children before exposure to viruses could be effective for the primary prevention of T1D. Antiviral drugs targeting viral or host proteins could be administered to susceptible individuals already exposed to multiple, recurrent or persistent infections in order to prevent or reduce the risk of appearance of islet autoimmunity and/or preserve β-cell function among newly and established diagnosed T1D patients. Alternative strategies targeting miRNAs, endogenous factors or based on natural products should be explored to fight EVs infections.

In conclusion, fighting EVs, especially CVB is possible. Vaccines are now being developed and offer hope for the prevention of T1D induced or aggravated by EVs, in particular by CVB. Various approaches to inhibit EVs infection, especially CVB, have been reported (Figure 1). However, the development of safe and effective antiviral drugs against CVB without generating drug-resistant variants remains a challenge. Therefore, to overcome drug resistance, antiviral strategies based on combination of antivirals and molecules targeting host proteins will be needed [94,95,96]. In addition, strategies targeting miRNAs and other endogenous factors or based on natural products such as bacterial compounds or plant extracts should be explored to fight against EVs infections and their effects on the host in order to prevent and/or treat T1D.

## Data Availability

Not applicable.
